# Comparing the efficacy of transmucosal and subcutaneous glucagon administration in response to insulin-induced hypoglycemia in healthy cats

**DOI:** 10.1177/1098612X261471152

**Published:** 2026-07-13

**Authors:** Jessica Pui Man Lam, Olivia Justus-Heale, Elisabeth Snead

**Affiliations:** 1 Internal Medicine Department, Department of Small Animal Clinical Sciences, Western College of Veterinary Medicine, University of Saskatchewan, Saskatoon, SK, Canada

**Keywords:** Glucagon, hypoglycemia, insulin overdose, diabetes mellitus, serum potassium, intranasal administration, subcutaneous administration, rectal administration

## Abstract

**Objectives:**

The aim of the present study was to evaluate the efficacy of two different formulations of glucagon given by three different routes (subcutaneous [SC], rectal [RCT] and intranasal [IN]) for correcting insulin-induced hypoglycemia in cats and to describe any adverse effects and compare the time required for administration by each route.

**Methods:**

A randomized, non-blinded, sham-controlled crossover study was conducted in six healthy cats, with a 7-day washout period between treatments. Hypoglycemia was induced using a constant rate infusion (CRI) of rapid-acting insulin lispro. Glucagon nasal powder (single-dose, ready-to-use) was given intranasally and rectally and compared with SC glucagon injection and IN sham control. Blood glucose (BG) concentration was measured from samples collected at baseline (T–15), after induction of hypoglycemia (T0) and at 5-min intervals for 30 mins, and then at 15-min intervals for 1 h after glucagon administration. Plasma glucagon and potassium (K^+^) concentrations were measured at T–15, T0, T15 and T30 after glucagon administration. The time taken to prepare and administer each treatment was recorded.

**Results:**

Median BG was 2.6 mmol/l at T0 and increased significantly after glucagon administration by all routes, reaching peak median concentrations of 11.2 mmol/l (IN), 9.7 mmol/l (RCT) and 10.15 mmol/l (SC). Plasma glucagon concentrations increased markedly by T15, with the highest concentrations observed after IN administration (1562 pmol/l), followed by SC (191.7 pmol/l) and RCT (158.5 pmol/l) routes of administration. Plasma K^^+^^ decreased after insulin administration and increased modestly by T30 after glucagon administration by all routes. No serious adverse effects occurred after glucagon administration.

**Conclusions and relevance:**

Transmucosal glucagon effectively restores BG towards baseline after insulin-induced hypoglycemia with minimal side effects. The IN route resulted in the highest plasma glucagon and BG concentrations compared with the RCT and SC routes of administration. Further studies are needed to quantify the efficacy and safety of transmucosal glucagon in diabetic cats in response to spontaneous insulin-induced hypoglycemia.

## Plain language summary

Comparing two ways of delivering glucagon to rescue cats with low blood sugar

The aim of the study was to compare different formulations of glucagon (a hormone drug) administered in three different ways: under the skin, rectally or intranasally. Six healthy cats received an intravenous insulin infusion to gradually and safely induce hypoglycemia. Once the target blood glucose level was reached, glucagon was administered by one of the three routes, or air was administered as a placebo using the nasal device. Blood samples were collected to monitor blood glucose, electrolyte levels and drug concentrations. Side effects and the time required to administer the drug were also recorded. Regardless of whether the drug was administered under the skin, rectally or intranasally, the average blood glucose level increased after administration, rescuing the cats from hypoglycemia within 10 mins. Drug absorption was highest with intranasal administration, followed by subcutaneous and rectal administration. One electrolyte level decreased when the cats became hypoglycemic, but administration of the drug did not worsen this change. No major side effects were observed after drug administration. The new methods of delivering glucagon intranasally or rectally successfully rescued cats from hypoglycemia. However, further studies are needed in diabetic cats experiencing hypoglycemia due to insulin overdose.

## Introduction

Insulin-induced hypoglycemia (IIH) remains one of the most significant challenges in the management of diabetes mellitus in both human and veterinary patients.^1,2^ In diabetic individuals, the normal physiologic secretion of glucagon in response to IIH is often impaired, making them susceptible to life-threatening hypoglycemic episodes.^1,2^

Glucagon, a key counter-regulatory hormone, functions by promoting hepatic glycogenolysis, gluconeogenesis and by suppressing insulin secretion to raise circulating glucose concentrations.^1^ In human medicine, glucagon is commonly administered as an emergency intervention for IIH, particularly in situations where oral carbohydrate administration is not possible or advisable, such as with impaired mentation (disorientation, seizures, coma) or if the patient is vomiting.^3^ Injectable formulations of glucagon require reconstitution immediately before administration.^4^ In humans, studies have shown that only 13% of caregivers were able to correctly administer a full dose of injectable glucagon in simulated emergency scenarios, highlighting the need for simpler delivery systems.^5^ Glucagon nasal powder (Baqsimi; Amphastar Pharmaceuticals) offers a premeasured, fixed (3 mg) dose in a ready-to-use, single-dose container for administration via a nostril.

In cats, injectable glucagon – particularly by the intravenous (IV) route – has been associated with adverse effects, such as vomiting, which poses additional risks in neurologically compromised or obtunded hypoglycemic patients.^6^ Cohen et al^7^ evaluated the efficacy of transmucosal glucagon administration in healthy euglycemic cats and demonstrated that both intranasal (IN) and rectal (RCT) routes significantly increased plasma glucagon and whole blood glucose (BG) concentrations within 15 mins of administration compared with a sham. IN glucagon was well tolerated, with mild and self-limiting adverse effects.^7^ However, to date, there are no published data confirming the efficacy of glucagon powder administered intranasally or rectally in cats with IIH.

The objectives of this study were to assess and compare the efficacy (increase in BG), safety (adverse effects) and preparation time of glucagon administration by IN, RCT and subcutaneous (SC) routes in healthy cats with experimentally induced hypoglycemia.

## Materials and methods

### Study design

A randomized, sham-controlled, non-blinded, crossover study was conducted in healthy research cats. Each cat was randomly assigned to receive sham or glucagon by three different administration routes (IN, RCT or SC) in a rotation sequence over a 4-week period with a 7-day washout period between treatments.

### Animals

Six domestic shorthair research cats (three spayed females, three castrated males), aged 2–5 years, were utilized. All cats were deemed healthy based on an unremarkable medical history, physical examination, systemic blood pressure measurement, complete blood count (CBC), serum biochemistry and urinalysis. An automated CBC was performed by Prairie Diagnostic Services (PDS) in Saskatoon, Saskatchewan, Canada, using the ProCyte Dx Hematology Analyzer (IDEXX Laboratories) and a single investigator (JPML) manually evaluated all blood smears. The serum biochemistry panels were performed using a Catalyst One Chemistry Analyzer (IDEXX Laboratories). For the urinalysis, a refractometer was used to measure the urine specific gravity and a urine dipstick (Chemstrip 9; Roche Diagnostics) was used for chemical analysis. Sediment analysis was performed from a wet mount of the urine sediment after centrifugation. Non-invasive indirect blood pressure (NIBP) was measured with a high definition oscillometric device (S+B medVET) following American College of Veterinary Internal Medicine guidelines.^8^ A single investigator (JPML) recorded NIBP after a 15-min acclimation period in a quiet room, using the same cuff and location (tail) for each cat. When not being tested, cats were co-housed in compatible groups and individually housed in a cage only during testing. The experiment and animal care protocol were approved by the Animal Care and Use Committee and the Institutional Review Board of the Western College of Veterinary Medicine, University of Saskatchewan (number 20240076).

### Acclimation and handling protocol

During the first week, cats underwent daily sessions that included full physical examinations and gentle handling for 10–15 mins per session to acclimate them to restraint for sample collection and being gently restrained in a calming wrap (Calm and Cozy Vet Products). Positive reinforcement using treats and toys were incorporated. Low-stress handling techniques were consistently applied to reduce anxiety and increase familiarity with experimental procedures.

### Drug and sham administration

For all glucagon and sham administrations, cats were gently restrained. Glucagon 3 mg nasal powder (Baqsimi; Amphastar Pharmaceuticals) was used for IN and RCT administration. For IN glucagon and sham treatments, the applicator tip was positioned against one nostril to form a seal and the plunger was depressed according to the manufacturer’s instructions (see S1 in the supplementary material). For RCT administration, the applicator tip was fully inserted into the rectum until only the plunger remained visible. With the applicator tip in place, the anus was pinched creating a seal around the application tip before depressing the plunger. The sham treatment was carried out using an empty glucagon nasal powder single-dose container that had been thoroughly washed, cleaned, dried and filled with 6 ml of air. Because of the single-use nature of the glucagon nasal powder container, it was not possible to load an inert powder to more accurately simulate the carrier powder for the sham experience.

For the SC route, lyophilized glucagon powder (1 mg) was reconstituted with the provided 1 ml of sterile diluent (Glucagon Emergency Kit; Eli Lilly). The vial was swirled gently until glucagon dissolved completely. The entire 1 ml resulting glucagon solution was then injected subcutaneously in the loose skin on the lateral thorax. Gentle pressure was applied at the injection site after withdrawal of the needle.

### Experimental set-up and animal preparation

Before each intervention, two peripheral IV catheters were placed the day before and food was withheld for 10–12 h, with water available ad libitum until the first sample collection. For IV catheter placement, sedation with dexmedetomidine (0.005 mg/kg IM) and application of lidocaine 5% cream (Maxilene 5; RGR Pharma) on the shaved skin was used to minimize pain and discomfort. Peripheral IV catheters (20–25 G) were aseptically placed in both cephalic veins, one for administration of a constant rate infusion (CRI) of insulin and the other to allow for blood sampling. Sedation was reversed using atipamezole (0.005 mg/kg IM). If an IV catheter was removed overnight or had to be replaced on the morning of the experiment, a minimum of 1 h was allowed between administration of the reversal agent and the start of experiment. Gabapentin capsules (100 mg, Teva-Gabapentin; Teva Canada) were administered orally the night before testing (minimum 10 h before the first sample collection) to help reduce overnight stress due to housing in kennels outside the animal care unit. Before each intervention, a pre-procedure physical examination, including measurement of baseline vital parameters (temperature, pulse, respiration [TPR]) and NIBP was performed with values recorded.

### Insulin infusion and hypoglycemia induction

Hypoglycemia was induced using a CRI of insulin lispro (100 μ/ml, Humalog; Eli Lilly), an ultra-short-acting insulin, with serial glucose monitoring until target hypoglycemia (T0) was achieved. Owing to both glucagon administration and hypoglycemia being associated with nausea, maropitant (1 mg/kg IV, Emavert; Dechra) was administered 30 mins before starting the insulin CRI. The insulin CRI was initiated at 0.09–0.10 U/kg/h through one peripheral catheter, and BG using whole blood was monitored every 5 mins using a glucometer (AlphaTrak3) calibrated for use in cats, with blood sampled from a peripheral ear vein for the first 30 mins. After the first 30 mins, the CRI rate was left unchanged if the BG was steadily decreasing, but if the response was minimal, the infusion rate was increased by 0.01–0.02 U/kg/h and maintained for another 30 mins. This was repeated until the target BG of mild IIH (2.8–3.0 mmol/l [50–54 mg/dl]) was reached based on the AlphaTrak3 reading. At this stage, the insulin CRI was discontinued, the IV line removed and the CRI catheter flushed with 2 ml of sterile 0.9% saline. All vital parameters were assessed; after 2 mins, a whole blood sample was collected for the T0 time point for determination of a plasma glucagon, potassium (K^^+^^) and serum glucose.

### Blood sampling

For each experiment, whole BG concentrations were measured using both a glucometer (AlphaTrak3) in real time as well as by collection of a blood sample for determination of serum BG using a more accurate hexokinase assay at specific time points outlined in Figure 1 from the sampling IV catheter. For serum BG determination, blood was collected into serum tubes (Vacutainers plus; BD Biosciences); once clotted, the sample was centrifuged at 1500 *g* for 10 mins and the serum harvested and stored at −80°C. Serum BG samples were measured by PDS using a Cobas C311 (Roche Diagnostics) analyzer. Point-of-care whole blood K^+^ measurement was performed using an enterprise point-of-care (EPOC) (Siemens Healthcare) blood gas machine. For plasma glucagon, blood samples were collected into chilled EDTA tubes (pAVPEDTA; BD Biosciences) at the time points outlined in Figure 1. Samples were immediately centrifuged at 4°C for 10 mins at 1500 *g* and the plasma was harvested and immediately frozen and stored at −80°C. Glucagon concentrations in plasma were measured by a commercial ELISA using an alternative sequential protocol validated for use in cats (Mercodia AB, catalogue number 10-1281-01).^6,9^ The assays were performed by Eve Technologies, according to the manufacturer’s instructions. Product information stipulates an assay sensitivity of 1.0 pmol/ml with a detection range of 1.5–130 pmol/l. All standards, controls and samples were run in duplicate. For all measurements, the optical density was generated from each well using a plate reader capable of reading at 450 nm. The mean intra-assay coefficient of variation (CV) was determined by using all glucagon samples run in duplicate on a single ELISA plate. The mean inter-assay CV was determined by using the glucagon concentration in a plasma sample from an arbitrary cat repeated on all plates.

**Figure 1:**
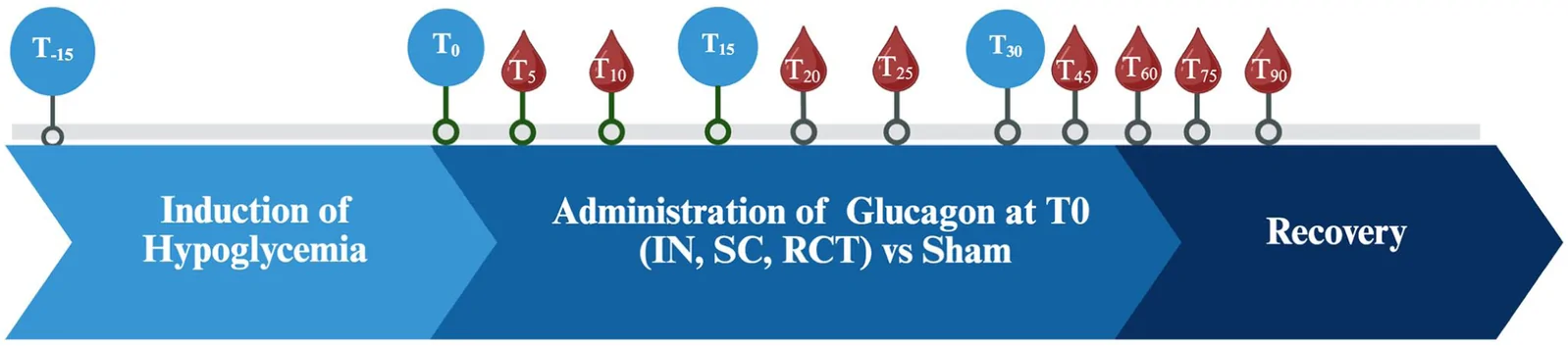
Visual representation of sampling time points and the experimental protocol. Hypoglycemia was induced in cats using an ultrashort-acting insulin lispro intravenous constant rate infusion with continuous glucose monitoring until target hypoglycemia (T0) was achieved. At T0, cats received glucagon via intranasal (IN), rectal (RCT) or subcutaneous (SC) administration, or sham based on a random order generator. Blood samples were collected at baseline (T–15) and serially after treatment (T0–T90) for measurement of glucagon concentration from plasma and potassium concentration from whole blood (blue circle time points) and blood glucose (BG) with glucometer (AlphaTrak3; Zoetis) (red time points). Cats were monitored for clinical signs and adverse effects throughout the study. The primary endpoint was achievement of BG ⩾4.1 mmol/l (74 mg/dl); the secondary endpoint was an increase in BG of ⩾1.1 mmol/l (20 mg/dl) above the nadir in an individual cat. Created in BioRender, Jessica Lam (2026) https://BioRender.com/1crstku

### Glucagon administration and blood sampling

Glucagon was administered to each cat by the specified route based on the predetermined randomized order. The time from preparation of the product to completion of administration was recorded for all routes. Figure 1 shows the time of sampling for BG, potassium and glucagon.

### Outcome measures and monitoring

The primary outcome was restoration of euglycemia, defined as either achievement of a BG ⩾4.1 mmol/l (74 mg/dl) (primary endpoint) or an increase of ⩾1.1 mmol/l (20 mg/dl) above baseline (secondary endpoint). If this was not met by 30 mins after treatment, or severe clinical signs of IIH occurred, a rescue dose of diluted 50% dextrose (0.5–1 ml/kg dextrose 50%) was available to be administered slowly via the CRI IV catheter. If such rescue was administered, monitoring continued but the glucagon treatment was considered a failure.

After glucagon or sham administration, monitoring included serial TPR, systolic blood pressure and mentation assessments every 5–15 mins until recovery. Adverse effects associated with glucagon administration, total blood sample volume collected, total flush volume and rescue therapy use, and time to euglycemia were documented on standardized data collection forms. Cats were gently restrained for the duration of the test until euglycemia was documented and then housed individually in a kennel for an additional 4 h to ensure their mentation and all vitals remained stable. Food was offered within 2 h of recovery from IIH. Further monitoring of adverse effects continued daily for all cats while group housed between tests.

### Statistical analysis

Statistical analyses and graphs were performed using a commercial software package (GraphPad PRISM10 software version 10.4.1). All data are presented as medians with ranges. Non-parametric repeated measures analyses were employed for all comparisons. Results from time points T–15, T0, T15 and T30 were compared within each treatment group (IN, RCT and SC) for serum BG, and plasma K^+^ and glucagon concentrations using Friedman tests, with adjusted *P* values reported from Dunn’s multiple comparison tests.

Peak concentrations for each analyte (serum BG, plasma K and glucagon) were defined as the difference between the maximum concentration after glucagon administration and the lowest value before glucagon administration. The peak concentrations were compared between groups using Friedman’s test with Dunn’s post-hoc analysis, reporting adjusted *P* values. Statistical significance for all tests was defined as *P* <0.05. Glucagon values were represented in natural log because of the wide ranges of values.

## Results

### Induction of hypoglycemia

Three castrated males and three spayed females were included in the study, with a median age of 3.4 years (range 2–5) and median body weight of 4.3 kg (range 3.7–5.8). An initial CRI rate of 0.09 units/kg/h of insulin lispro was utilized for all cats, and the median dose of insulin lispro required to achieve the hypoglycemic target of 2.8 mmol/l (50–54 mg/dl) was 0.13 U/kg/h, with a total dose of 1.3 U/cat. A total of 24 episodes of hypoglycemia were induced, with a median time to achieve the hypoglycemic target of 2.8 mmol/l (50–54 mg/dl) of 120 mins (range 64–220). In 19/24 hypoglycemic events, there was a significant drop in median heart rate from 140 bpm (range 120–180) at baseline (T–15) to 120 bpm (range 100–160) at T0 (*P* <0.001). As hypoglycemia progressed, the mentation of all cats shifted from alert and responsive to listless and lethargic, which was also accompanied by hypersensitivity to noises.

### Drug application and tolerability

Of the six cats in the SC treatment group, two (cats 2 and 4) moved at the time of injection, leading to approximately 0.1 ml of the 1 ml glucagon solution leaking from the injection site. Despite minor leakage of solution in the SC group, increases in BG and glucagon concentration were observed, suggesting the sufficient dose was still delivered to achieve physiologic response. All six cats receiving the glucagon nasal glucagon (IN and RCT) treatments were compliant and received the treatment without struggle. Of the six cats in the IN glucagon group, two (cats 2 and 3) had powder blocking their small nostrils after administration and expelled some powder after administration. Despite this, the BG and glucagon concentrations both increased significantly from T0, documenting successful absorption of a sufficient dose of the IN glucagon.

Adverse effects associated with administration of glucagon for each route and the sham are reported in Table 1. Sneezing was the most common adverse effects for cats receiving IN glucagon (n = 6/6), followed by lip smacking (n = 3/6), nasal discharge (n = 2/6), hypersalivation and blepharospasm (n = 1/6).

**Table 1:** Summary of adverse effects observed between different treatments (n = 6) for each treatment

Treatment	Nasal discharge	Sneezing	Hypersalivation	Vomiting	Lip smacking	Skin sensitivity	Blepharospasm
SC	0	0	0	0	0	2	0
RCT	0	0	0	0	0	0	2
IN	2	6	1	0	3	0	1
Sham	0	0	0	0	0	0	1

IN = intranasal; RCT = rectal; SC = subcutaneous

### Primary vs secondary time points

There was no significant difference between the time to reach the primary (BG ⩾4.1 mmol/l [74 mg/dl]) or secondary (BG ⩾1.1 mmol/l [20 mg/dl] increase from T0) end points with administration of glucagon by the different routes (Table 2).

**Table 2:** Time (mins) to reach the primary endpoint (blood glucose [BG] ⩾4.1 mmol/; [74 mg/dl]) and the secondary endpoint (increase in blood glucose ⩾1.1 mmol/L [20 mg/dl] from T0) after glucagon administration via different routes

Route	Primary endpoint: median time (mins) to BG (⩾4.1 mmol/l)	Secondary endpoint: median time (mins) to BG (↑ ⩾1.1 mmol/l)	*P* value
SC	8.3 (5–15)	7.5 (5–15)	>0.99
RCT	5.0 (5–5)	5.0 (5–5)	1
IN	7.5 (5–10)	7.5 (5–10)	1
Sham	49 (25–90)	40 (25–60)	0.25

Data are median (interquartile range). *P* values represent within-route comparisons between primary and secondary endpoint times (paired analysis). A *P* value <0.05 was considered statistically significant

IN = intranasal; RCT = rectal; SC = subcutaneous

### Drug efficacy

Whole blood K^+^ concentration was available for all time points except for cat 1 at T15 (SC, IN glucagon, sham) and T30 (SC), cat 3 at T15 (SC) and cat 4 at T15 (SC) and T30 (RCT) due to a malfunction of the EPOC analyzer. Serum K^+^ concentrations for those missing time points were determined by PDS.

The overall median baseline (T–15) serum BG concentration was 5.63 mmol/l (range 5.4–6.8; 101 mg/dl [range 97–122]). The median hypoglycemic serum BG at T0 was 2.6 mmol/l (range 2.2–3.0; 47 mg/dl [range 40–54]) across all groups. BG concentrations increased in the SC, IN and RCT glucagon treatment groups as early as 5 mins after administration (Figures 2 and 3). The maximum increase in BG was observed at 20 mins, with median values of 11.2 mmol/l (range 10.3–15.2; 202 mg/dl [range 185–274]), 10.15 mmol/l (range 8.2–10.8; 182.7 mg/dl [range 148–194]) and 9.7 mmol/l (range 8.7–12.2; 174.6 mg/dl [range 157–220]) for the IN, SC and RCT groups, respectively. Compared with the sham group, which had a recovery median of 5.1 mmol/l (range 3.3–5.7; 92 mg/dl [range 59–103]) at 60 mins, the glucagon groups showed significantly faster recovery and higher peak BG concentrations (*P* = 0.03).

**Figure 2:**
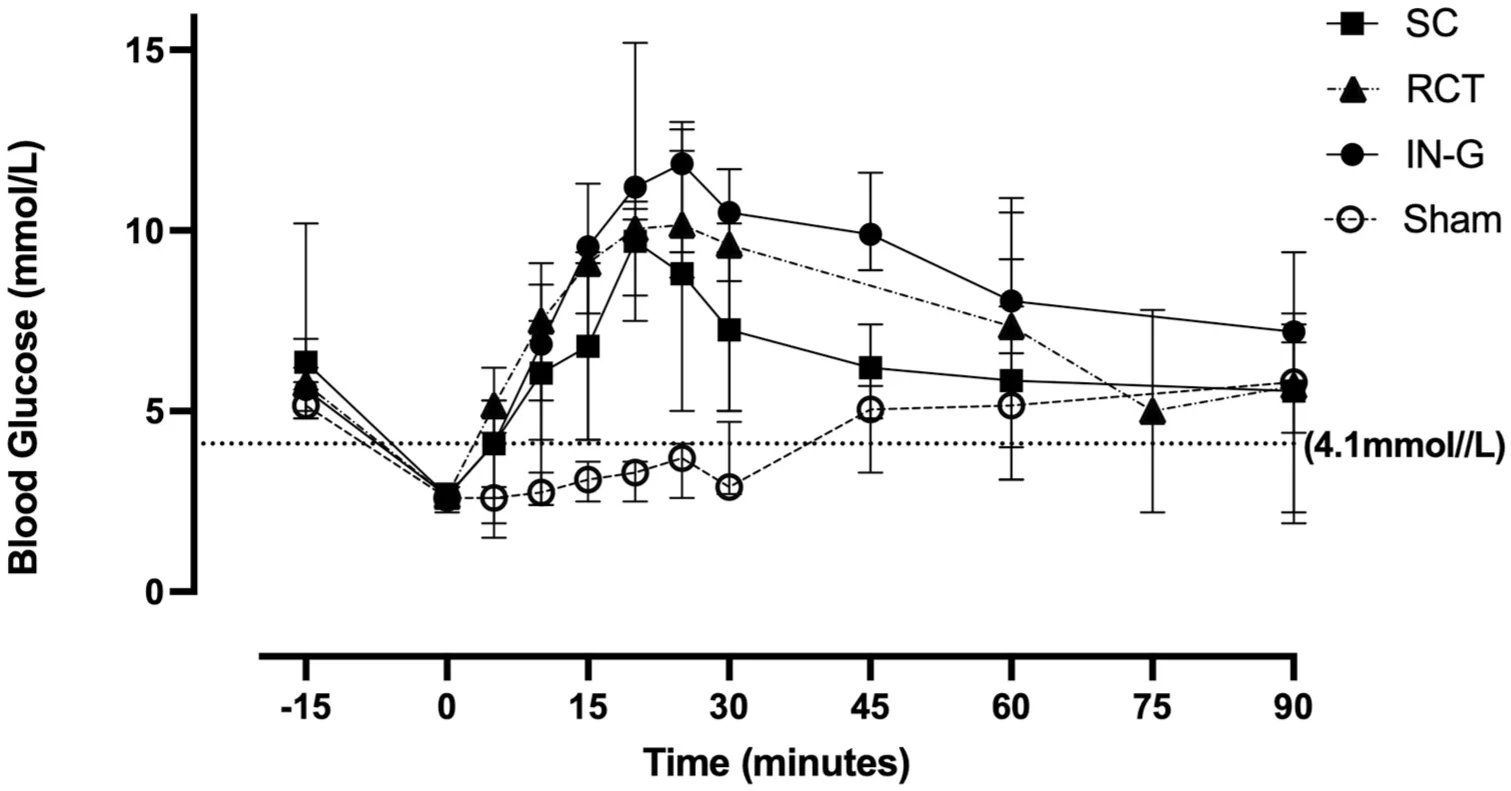
Median and ranges of blood glucose concentrations before and after insulin-induced hypoglycemia for the subcutaneous (SC) glucagon group (black square), intranasal glucagon (IN-G) group (black circle), sham group (open circle) and rectal (RCT) glucagon group (black triangle)

**Figure 3:**
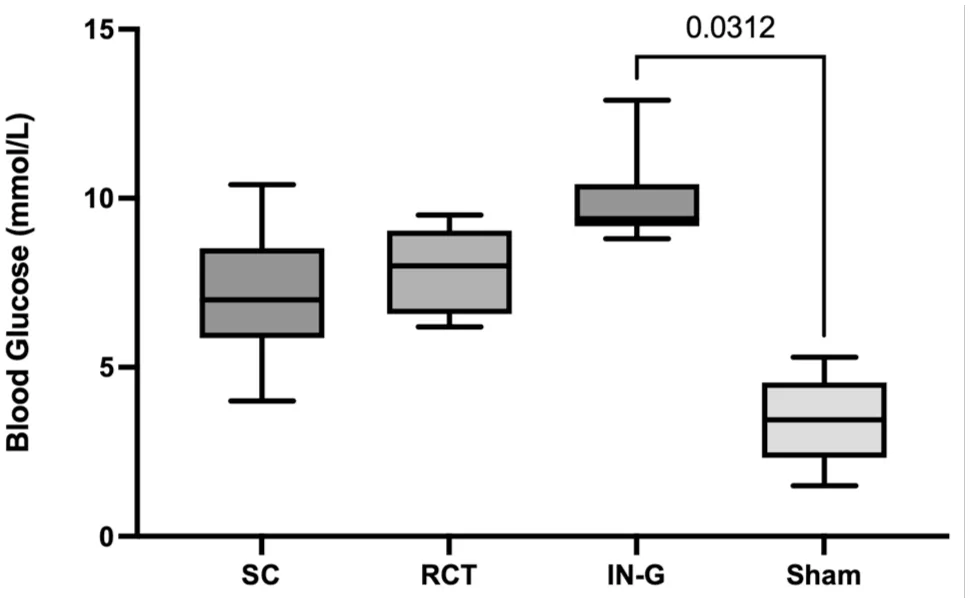
Box and whisker plot of maximum increase of blood glucose (BG) concentration in six cats after glucagon administration. The top and bottom whiskers represent maximum and minimum values of the group, respectively. There was a significant increase in BG concentration when glucagon was given intranasally compared with intranasal sham. RCT = rectal; SC = subcutaneous; IN-G = intranasal glucagon

The median plasma glucagon measured at the set time points for all four treatment groups are shown in Figure 4 and Table 3.

**Figure 4:**
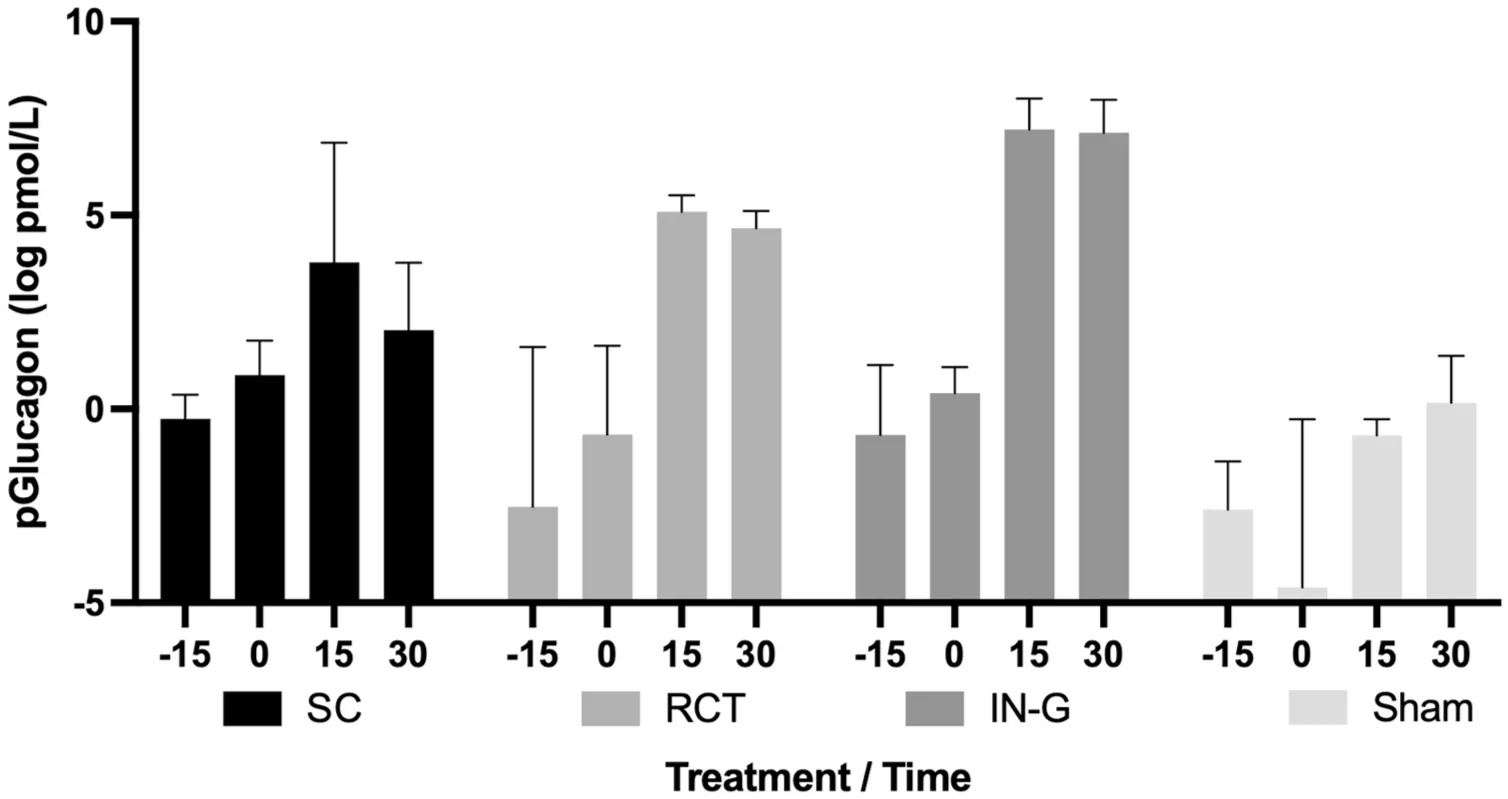
Log of median values and ranges for plasma glucagon (pGlucagon) concentration in the subcutaneous (SC) glucagon group, rectal (RCT) glucagon group, intranasal glucagon (IN-G) group and sham group

**Table 3:** Glucagon concentrations (pmol/l) at set time points (T–15, T0, T15 and T30) with *P* values for within-group comparisons to T0 (time of hypoglycemia and before treatment administration)

Treatment	T–15	T0	*P* (T–15 vs T0)	T15	*P* (T15 vs T0)	T30	*P* (T30 vs T0)
SC	0.8 (0.14–1.3)	2.617 (0.26–5.8)	0.79	191.7	0.001	12.89 (0.51–44.77)	0.35
RCT	1.37 (0.01–4.95)	1.569 (0.01–5.1)	>0.99	158.5 (39.4–249)	0.004	110 (26–164.9)	0.06
IN	1.02 (0.01–3.1)	1.105 (0.01–2.96)	>0.99	1562 (403–3000)	0.01	1539 (413–2908)	0.01
Sham	0.10 (0.01–0.26)	0.05 (0.01–0.26)	>0.99	0.47 (0.01–0.77)	0.44	1.34 (0.01–3.95)	0.22

Data are median (range). A *P* value <0.05 is considered statistically significant

IN = intranasal; RCT = rectal; SC = subcutaneous

When comparing between-treatment groups, the maximum change in concentration of glucagon from T0 based on the sampling time points was 189 pmol/l (range 1.5–959), 1725 pmol/l (range 684–2997), 159 pmol/l (range 34–249) and 1.2 pmol/l (range 0–3.9) in the SC, IN, RCT and sham groups, respectively. The peak glucagon concentration for the IN glucagon group was statistically higher than in the SC (*P* = 0.0045) and sham groups (*P* = 0.0312) but was not different from the RCT group (*P* = 0. 0471). The RCT glucagon group was statistically higher than the sham group (*P* = 0.04), whereas the SC group did not differ significantly from the sham group (*P* = 0.35) (Figure 5).

**Figure 5:**
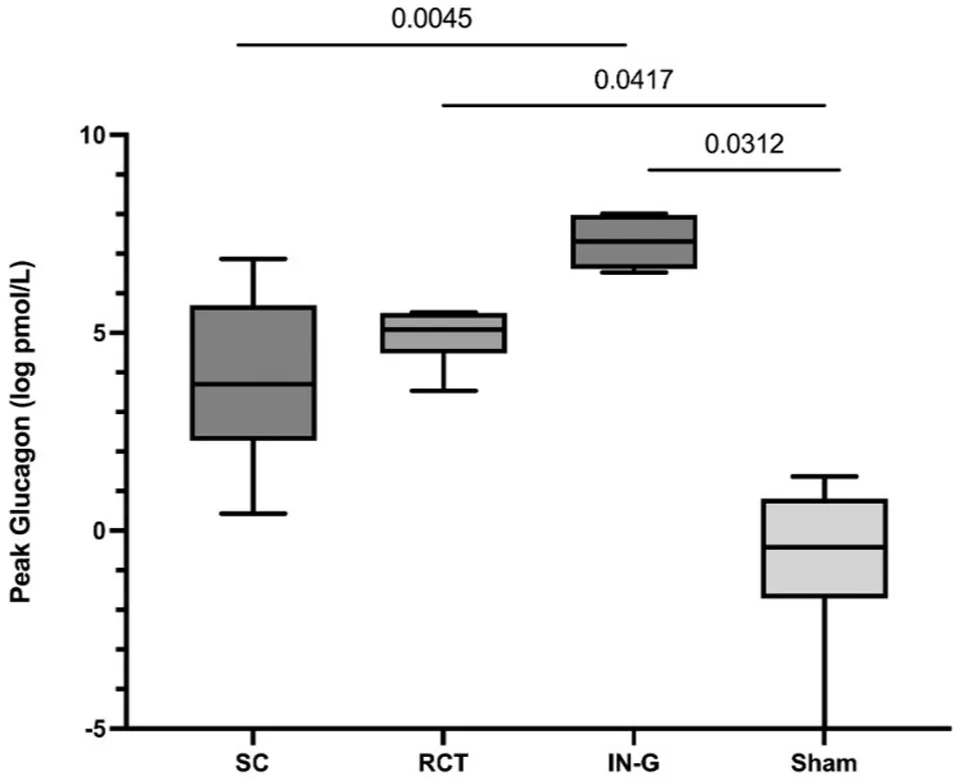
Box and whisker plot of log of maximum increase of plasma glucagon in six cats after glucagon administration. The top and bottom whiskers represent maximum and minimum values of the group respectively. There was significant increase in glucagon when given intranasally compared with sham. IN-G = intranasal glucagon; RCT = rectal; SC = subcutaneous

Table 4 summarizes the changes in median whole blood K^+^ concentration over time for each treatment. Across all groups, T–15 K^+^ was similar, with a median K^+^ of 3.8 mmol/l. Induction of hypoglycemia was associated with a significant decrease in K^+^ at T0, with a median of 2.8 mmol/l. By T30, the K^+^ had not returned to baseline but had increased from T0 for all treatment groups. There were no significant differences in K^+^ concentration at any of the time points between the four groups (Figure 6).

**Table 4:** Potassium between each treatment group at different time points before (T–15) and 15–30 mins after glucagon administration (T0)

Treatment	T–15	T0	T15	T30
SC	3.8 (3.4–4.1)	2.6 (2.5–3.0)	3.2 (2.6–4.3)	3.1 (2.8–3.3)
RCT	3.7 (3.5–3.8)	2.7 (2.1–3.0)	2.8 (2.2–3.0)	3.0 (2.4–3.4)
IN	3.8 (3.4–4.1)	2.8 (2.4–3.0)	2.8 (2.4–3.2)	3.1 (2.9–3.9)
Sham	3.8 (3.4–4.1)	2.8 (2.2–3.1)	3.0 (2.7–3.5)	3.0 (2.4–3.9)

Data are median (range)

IN = intranasal; RCT = rectal; SC = subcutaneous

**Figure 6:**
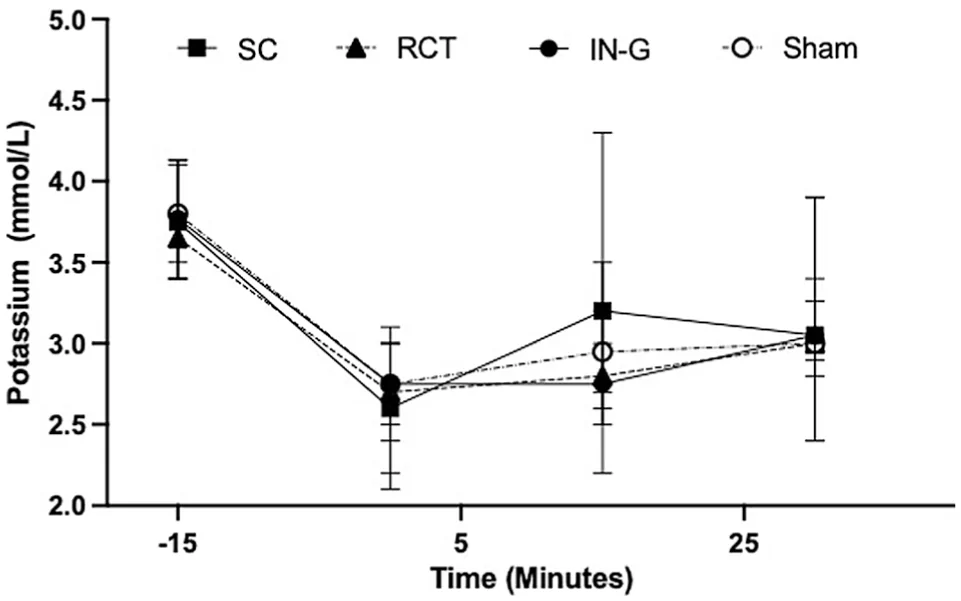
Median and ranges of plasma potassium concentration before insulin-induced hypoglycemia and after glucagon administration for the subcutaneous (SC) glucagon group (black square), intranasal glucagon (IN-G) (black circle), sham (open circle) and rectal (RCT) group (black triangle)

The median time for preparation (reconstitution) and administration for glucagon given by the IN or RCT route was significantly shorter (*P* = 0.002) than for the SC glucagon formulation – that is, 14 s (range 8–20) and 43 s (range 15–58), respectively (Figure 7).

**Figure 7:**
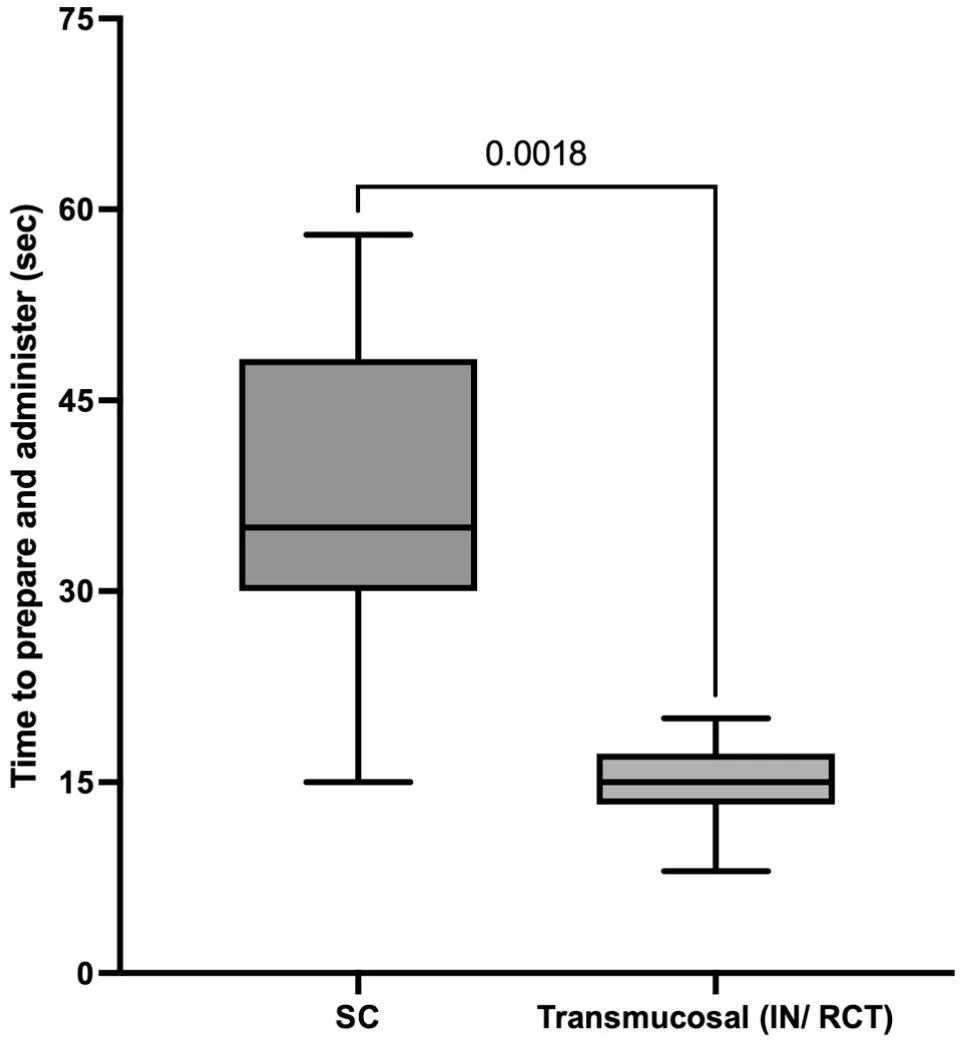
Box and whisker plot of time to prepare and administer subcutaneous (SC) lyophilized glucagon compared with transmucosal glucagon (intranasal [IN] and rectal [RCT]) groups. The top and bottom whiskers represent maximum and minimum values of the group, respectively. There was significantly shorter time to administer transmucosal glucagon compared with subcutaneous glucagon

## Discussion

This study demonstrates that glucagon nasal powder, administered transmucosally (IN or RCT), effectively increases BG concentrations when given in response to IIH in cats. The time to increase BG for glucagon nasal powder given intranasally or rectally and for SC administration of a lyophilized glucagon were comparable to times previously reported for IV and intramuscular (IM) glucagon administration, with BG peaking around 20–25 mins after administration.^6,7^ Administration by either route resolved the hypoglycemia with normoglycemia (primary endpoint of 4.1 mmol/l) being achieved within 10 mins. The secondary endpoint, time to reach a BG greater than or equal to 1.1 mmol/l from the nadir at T0, which was determined in case there were individuals with a slower recovery, was not applicable as all cats who received glucagon recovered past the primary endpoint within 10 mins.

Interestingly, although one previous study suggested that RCT glucagon powder resulted in greater increases in BG and higher glucagon absorption, our findings indicate that the IN route produced the highest post-administration plasma glucagon concentrations compared with either the SC or RCT routes.^7^ This discrepancy may be due to factors such as fecal material affecting RCT absorption in some cats or differences in the vehicle for absorption (transmucosal vs SC formulations). Challenges in medication administration due to patient movement were noted in our study with SC administration and may affect drug delivery, similar to findings from an earlier study using IN administration.^7^ Notably, the glucagon concentrations observed in this study were significantly higher, with median concentration of 1562 pmol/l after IN administration compared with only 158.5 pmol/l after RCT, and compared with 260 pmol/l and 349 pmol/l in a prior study for IN and RCT routes, respectively.^7^ Enhanced absorption in our study may be attributed to better subject compliance, and variance in how the assay was performed

After SC glucagon administration, glucagon concentration in the plasma returned to a baseline (T–15) concentration after 30 mins, while with the IN and RCT routes the elevated glucagon concentration was maintained at T30. The more rapid decline in glucagon concentration in plasma after SC administration was similar to that observed after IM injection in cats in another study, assuming first order kinetics.^6^ Our study offers valuable insights into both BG and glucagon dynamics after induction of IIH. In the sham group, glucagon concentration in plasma decreased in response to IIH, aligning with findings by Mott et al.^10^ In other species (in dogs and humans), sustained hypoglycemia for 270 mins has been required to activate endogenous glucagon secretion.^11^ It is possible that the duration of hypoglycemia in our study was too short to document a significant increase in endogenous glucagon secretion, as we aimed to induce only mild to moderate hypoglycemia.^10^

Doses used in this study reflected commercially available formulations for each administration route and were not adjusted for dose equivalence. This approach was chosen to evaluate the clinical performance of each glucagon product as used in practice, rather than pharmacological potency under standardized dosing. This design is consistent with human literature comparing marketed glucagon products and routes of administration, where real-world use rather than dose-normalized equivalence was assessed.^12,13^

The dull mentation observed after induction of hypoglycemia may have facilitated easier administration in the cats in our study compared with those in the study by Mott et al.^10^ In humans, prompt and successful glucagon administration is critical in treating severe hypoglycemia, relying on the patients’ or caregivers’ ability to prepare and administer the correct dose. A study involving 136 caregivers found that 69% experienced difficulty preparing and then administering injectable formulations – from properly opening containers, drawing up and mixing with a diluent, and then drawing up the full correct dose.^14^ Only a small minority (6–31%) were able to correctly administer a full dose of lyophilized glucagon.^15^ This highlights the importance of addressing human factors in emergency glucagon administration. Notably, we documented that preparation of the glucagon nasal powder device was twice as fast compared with reconstitution of the lyophilized powder formulation for SC administration, even among trained personnel, and the difference would be expected to be even longer for untrained personnel. Despite training, complete administration of the dose via the SC route was not always successful, with 2/6 cats not receiving their full dose. SC administration was chosen for this study over IM administration because of the familiarity that diabetic cat owners would have with administering SC insulin. The lower glucagon concentration in plasma and slower response in the SC group may be attributable to reduced tissue perfusion or SC fat distribution at the injection site, which may have delayed glucagon absorption if absorption does not follow first order kinetics.

The frequency of adverse effects we observed with glucagon nasal powder administration was lower than that reported in studies where IV glucagon was given and in the previous transmucosal glucagon study in cats.^6,7^ In human trials, common adverse effects associated with glucagon nasal powder included nausea, headaches, vomiting and upper respiratory irritation.^16^ In the previous transmucosal study, vomiting, hypersalivation and sneezing were reported, although these were typically self-limiting.^7^ As vomiting poses a significant risk for both hypoglycemic cats and humans, especially in obtunded patients who cannot protect their airways, we administered an anti-nausea medication before inducing hypoglycemia. Although this may have contributed to a lower incidence of vomiting in our study, the use of anti-nausea medication was deemed necessary for ethical reasons. Lip smacking was observed in 3/6 cats in the IN glucagon group, which could indicate nausea or irritation from the powder entering their mouths. In human medicine, unconscious patients who receive glucagon are placed in the recovery position within 5–15 mins, or once they become more responsive, to reduce the risk of aspiration. By adapting this practice from human medicine, pet owners administering IN glucagon at home may help decrease the incidence of aspiration pneumonia. Owners could also be given a dose of injectable SC antiemetic or a transdermal gel to apply right after administering the glucagon for at-home use to help minimize the risk of vomiting leading to aspiration after glucagon administration. In hospital, IV maropitant would be ideal to give shortly after glucagon administration.

Hypokalemia and transient hyperkalemia have been reported as adverse effects in humans with glucagon administration.^16^ In healthy cats, no clinically significant decrease in K^+^ concentration was observed in response to glucagon without an induction of IIH.^7^ In this study, although hypoglycemia induced a decrease in K^+^ concentration, administration of glucagon did not further lower the K^+^ concentration within the period of monitoring. Despite the normalization of glycemic levels, K^^+^^ concentration remained decreased with only a partial correction in the time frame covered by our monitoring. Although no signs of hypokalemia were seen after treatment, future studies should consider extending K^+^ monitoring until the resolution of hypokalemia is confirmed to assess time to normalization. The findings highlight the importance of monitoring K^+^ concentrations in diabetic patients experiencing IIH and after glucagon administration, particularly when K^+^ concentrations are already markedly reduced secondary to insulin overdose.

In Canada, SC injectable glucagon costs approximately CAD$380, whereas nasal glucagon powder costs approximately CAD$120. Given the efficacy observed in this study, nasal glucagon powder may represent both a reliable and more affordable treatment option in Canada. However, the relative affordability and accessibility of nasal glucagon powder compared with injectable glucagon formulations are country- and healthcare system-specific, and costs may differ substantially in other markets. Although some pet owners may still be deterred by the cost of nasal glucagon powder, this expense may be considerably lower than the cost associated with emergency treatment for IIH. In Canada, even a short 12–24-h hospitalization for basic treatment and monitoring can exceed CAD$1000. Therefore, investing in nasal glucagon powder could represent a cost-effective preventive measure that may reduce the frequency of emergency visits. In addition, the ease of administration of transmucosal glucagon compared with subcutaneous glucagon may further enhance its practicality and appeal for pet owners.

Study limitations include a small sample size, which increases the risk of a type II error. To promote a calmer baseline state and reduce anticipatory stress associated with handling, gabapentin was administered; however, gabapentin has been reported to cause severe hypoglycemia in both diabetic and non-diabetic humans.^16^ In order to limit the potential impacts on our results, we ensured a minimum of 10 h between dosing and the start of each experiment.

Hypoglycemia was induced using fasting and an insulin CRI, rather than a traditional stepped hyperinsulinemic-hypoglycemic clamp, which may have introduced additional variability in the BG concentration.^12^ However, this approach mirrors the method used in human studies evaluating the response to glucagon preparations and more closely simulates the conditions diabetic cats may experience at home during IIH.^17^

Sampling time for glucagon concentration analysis was limited to 30 mins, which may not fully capture later-phase differences in glucagon exposure between administration routes. Finally, counterregulatory hormones were not measured, as has been performed in some human studies; therefore, differences in counterregulatory responses between treatment groups or in individual cats affecting our results cannot be excluded.

## Conclusions

This study demonstrated that administration of transmucosal glucagon powder can rapidly and effectively raise BG concentrations after induction of mild to moderate hypoglycemia in healthy cats with minimal side effects. IN administration resulted in higher glucagon concentrations in plasma and higher early peak BG values compared with SC and RCT routes; however, these differences were not statistically significant. Monitoring K^+^ concentration in diabetic cats treated for IIH is also important because of the potential effects of both insulin and glucagon on plasma K^+^ concentrations. The use of antiemetic medication may have reduced vomiting incidence in this study but would also be recommended, whenever possible, at the same time as or before glucagon administration in the hospital, and possibly at home to prevent any unnecessary complications

Given glucagon nasal powder’s potential as a preventive measure for treating a hypoglycemic crisis, larger studies are needed to quantify its efficacy and safety in diabetic cats with spontaneous IIH ranging in severity to better assess the clinical outcomes.

## Supplemental Material

S1 Use of nasal glucagon powder in an insulin-induced hypoglycemic cat.
