# Supplementary material for: Comparing the efficacy of transmucosal and subcutaneous glucagon administration in response to insulin-induced hypoglycemia in healthy cats
Source: J Feline Med Surg. 2026 Jul 13;28(9):1098612X261471152. doi: 10.1177/1098612X261471152 (PMC13724890; doi:10.1177/1098612X261471152)
Supplement: Supplementary file 1 [file sj-docx-1-jfm-10.1177_1098612X261471152.docx]

Supplemental 1: Use of nasal glucagon powder in a insulin induced hypoglycemic cat.
